# Application of manual aspiration thrombectomy in the treatment of deep vein thrombosis in cancer patients: Descriptive retrospective cohort study

**DOI:** 10.1371/journal.pone.0255539

**Published:** 2021-08-04

**Authors:** Eu Hyun Kim, Hae Giu Lee, Jung Suk Oh, Ho Jong Chun, Byung Gil Choi, Myung Ah Lee

**Affiliations:** 1 Department of Radiology, Seoul St. Mary’s Hospital, College of Medicine, The Catholic University of Korea, Seocho-Gu, Seoul, Republic of Korea; 2 Department of Radiology, St. Vincent’s Hospital, College of Medicine, The Catholic University of Korea, Suwon-si, Gyeonggi-do, Republic of Korea; 3 Division of Medical Oncology, Department of Internal Medicine, Seoul St. Mary’s Hospital, College of Medicine, The Catholic University of Korea, Seocho-Gu, Seoul, Republic of Korea; Ohio State University Wexner Medical Center Department of Surgery, UNITED STATES

## Abstract

**Objectives:**

To determine the outcomes and feasibility of endovascular treatment, mainly based on manual aspiration thrombectomy (MAT) with adjunctive percutaneous balloon angioplasty with or without stent deployment, for treatment of symptomatic ilio-femoral deep vein thrombosis (IFDVT) in cancer patients.

**Materials and methods:**

In this retrospective cohort study, 135 consecutive patients (56 men; mean age, 63 years; 149 limbs) with acute (n = 113; 83.7%) and subacute to chronic (n = 22; 16.3%) symptomatic IFDVT underwent MAT-based endovascular treatment. Among them, adjunctive balloon angioplasty and stent placement was performed in 94 patients. Technical and clinical success regarding stage and cause of DVT was assessed.

**Results:**

Technical success (complete thrombus removal without residual thrombus or stenosis) was achieved in 89.6%, and subjective symptom improvement was stated by 71.5% of treated patients. The primary patency rates were 88.1%, 81.6%, 76.0%, 74.1% and 69.1% at 1, 3, 6, 12, and 30 months, respectively. Recurrent IFDVT occurred in 19.3% (26/135) of patients, 0.79 cases per patients-years of follow up. According to the analysis by causes of IFDVT, recurrence rate was 19.3% (11/57), 21.2% (12/57), and 14.3% (3/21) in unknown, compression/invasion of the vein by cancerous mass, and May-Thurner syndrome groups, respectively (p = 0.798). No procedure-related complication developed.

**Conclusions:**

Endovascular treatment based on MAT is a feasible treatment option with favorable outcomes and minimal risk of complication in cancer patients with symptomatic IFDVT.

## Introduction

Cancer is one of the major risk factors for venous thromboembolism (VTE), which refers to deep vein thrombosis (DVT) and pulmonary thromboembolism (PTE) [1], with varying degree of risk depending on the primary site and histologic subtype [2]. In addition, coincidence of other common risk factors of VTE such as recent surgery, systemic chemotherapy, or immobilization is high in cancer patients. The pathogenesis of cancer-related VTE is known to be multifactorial, including malignancy-associated hypercoagulability, external compression by tumor, and venous invasion or tumor thrombosis [3].

In spite of the high incidence of VTE in cancer patients, anticoagulation therapy, which has been considered as conventional therapeutic option [4], is not always possible because the patients often have increased bleeding tendency [5,6]. Endovascular treatment of ilio-femoral DVT (IFDVT) is another option and it includes three categories: pharmacologic thrombolysis including systemic or catheter-directed thrombolysis (CDT), percutaneous mechanical thrombectomy (PMT), and pharmacomechanical thrombolysis [7]. Although endovascular intervention for IFDVT is of limited use and even sometimes considered to be contraindicated in cancer patients due to uncertain or short life expectancy, the patients can definitely benefit from the endovascular treatment such as manual aspiration thrombectomy (MAT), since large thrombus burden is reduced in a relatively short procedural time [8]. Prompt interventional treatment for symptomatic IFDVT is essential to even cancer patients with a life expectancy of about one year, as it can improve their quality of life, alleviate symptoms, and prevent potentially serious complications such as massive PTE [9,10]. In addition, another possible complication of IFDVT, post-thrombotic syndrome (PTS), can be avoided if they survive long enough [9,10]. Among many different endovascular treatment, MAT, a type of PMT in which a lot of thrombus can be removed through a large-bore catheter by applying negative pressure, is superior to other types of endovascular treatment of IFDVT in that it is cost-effective, simple, quick, readily available in clinical practice, and safe as it is free of bleeding risk [11,12].

Recently, a few data on benefits of interventional treatment of IFDVT in cancer patients have been published [13,14] one of which demonstrated non-inferior outcome of pharmacomechanical thrombolysis using Angiojet in acute symptomatic IFDVT in a small number of cancer patients compared to cancer-free patients [13]. However, the therapeutic outcomes of MAT in cancer patients with IFDVT have not been investigated, so this study was conducted to evaluate outcomes and feasibility of MAT, for symptomatic IFDVT in cancer patients.

## Materials and methods

### Ethics statement

This study was approved by the Institutional Review Board and Ethics Committee of Seoul St. Mary’s Hospital, College of Medicine, The Catholic University of Korea (approval number: KC18RESI0570). Written consent was specifically waived by the approving institutional review board because this study was based on a retrospective medical chart and image review. All data were fully anonymized before access them, and the EMR and PACS data of Seoul St. Mary’s Hospital patients for 13 years and 5 months included in the study were analyzed.

### Patients

Between February 2005 and May 2018, a total of 278 patients with symptomatic lower extremity IFDVT who were diagnosed by pre-procedural contrast-enhanced computed tomography (CECT) with or without color Doppler ultrasonography (CDUS) were managed with MAT. All patients underwent either CT pulmonary angiography with lower extremity CT venography, or abdomen and chest CT in addition to lower extremity CDUS. Among the 278 patients, 143 patients without underlying malignancy were excluded. Medical records, CECT and CEUS images, and lower extremity venography of the patients were reviewed. The indications for treatment were that 1) patients were expected to have a life expectancy of 6 months or more, and 2) had Eastern Cooperative Oncology Group (ECOG) performance status (PS) of 2 or greater, but patients who complained extreme symptoms and desired treatment for DVT were also included. The included patients were closely followed up for recurrent symptoms everyday while hospitalized. After discharge, most of the patients visited outpatient clinic every 3–4 weeks for cancer treatment as well as surveillance for recurrent VTE.

The extent and stage based on the symptom duration (acute: <14 days, subacute to chronic: ≥14 days) [7] were analyzed. In addition, the causes of IFDVT based on pre-procedural CECT were categorized as the following: 1) unknown (without discernible cause), 2) compression or invasion of the vein by cancerous mass, and 3) May-Thurner syndrome. The origin sites of malignancy were investigated as well as histopathology of the cancer. Occurrence of procedure-related complications was assessed. Reported risk factors related to VTE recurrence, including age, male sex, recent surgery, coagulopathy, immobilization, recent chemotherapy, hypertension, were investigated [15,16]. In addition, laboratory findings of D-dimer level and pre- and post-procedural hemoglobin (Hb) and hematocrit (Hct) were recorded.

### Procedures

The manual aspiration thrombectomy was implemented as mainstay therapy for patients with ilio-femoral DVT at the discretion of the treating physician at our institution during the study period. Based on the operator’s decision whether thrombus has risk of migration to develop PTE during the procedure, prophylactic transjugular or transfemoral IVC filter was placed at the beginning of the procedure. Heparin (5000 IU) was administered intravenously to all patients. Then, antegrade access of the ipsilateral popliteal vein on prone position (or femoral vein on supine position when only iliac vein was involved) was obtained using a 21-gauge micropuncture needle set (Cook, Bloomington, IN) under ultrasound-guidance. After placement of an 11 Fr vascular sheath (Terumo, Tokyo, Japan), an 8 Fr guiding catheter (Shuttle, Cook, Bloomington, IN) was introduced over a 0.035-inch hydrophilic guidewire (Terumo) and 20-mL syringe was connected to the catheter after removing the guidewire and dilator. MAT was performed by moving the catheter back and forth while applying negative pressure through the syringe. Intermittent ascending venography was performed to check for remaining thrombus burden and restoration of flow during the aspiration. The process was repeated several times until sufficient removal of thrombi. For management of the residual stenotic segment in the iliac vein despite attempts of thrombus aspiration, the hydrophilic guidewire was located crossing the stenosis and it was further dilated with percutaneous transluminal angioplasty (PTA) using a balloon catheter with or without deployment of self-expandable stent(s). Completion venography was performed to confirm continuous antegrade flow from the popliteal vein to the IVC without intraluminal filling defect and adjacent collaterals, as mentioned in the Korean practice guidelines [15]. In cases with residual stenosis or thrombus on venography, additional PTA was repetitively performed. After MAT, anticoagulation therapy either with Dalteparin (Fragmin®), low-molecular weight heparins (LMWH), or Rivaroxaban (Xarelto®), Oral Factor Xa Inhibitor, was started according to the opinion of oncologist, the attending physician.

### Definition of outcome parameters

Pre- and post-procedural venography was compared and degree of thrombus removal was evaluated whether remaining thombus with or without stenosis is present. Then, restoration of antegrade venous flow without residual thrombus was defined as technical success. Clinical success was defined as resolution of symptoms related to IFDVT, such as leg swelling, leg pain, and color change. Primary patency and complications were evaluated according to the Society of Interventional Radiology guidelines [7]. The definition of primary patency and recurrence of DVT were set in accordance with the Korean Practice Guidelines [15]. Primary patency is defined as preserved patency without any intervention after technically successful treatment. Recurrence of IFDVT was diagnosed by CDUS or CECT when symptom relapsed, and the diagnosis was confirmed with findings of non-compressibility of the common femoral or popliteal veins on CDUS or low density filling in the veins on CECT.

### Statistical analysis

Statistical analysis was performed using the Statistical Package for Social Sciences (IBM SPSS Statistics 24, Chicago, IL). Student t-test and chi-square test were used for comparison of continuous and categorical variables, respectively. Logistic regression was used to determine the independent risk factors. Primary patency was estimated by Kaplan-Meier method, and comparisons of patency according to cause were performed with log-rank test. The Cox proportional hazards model was used for the multivariable analysis to assess the influence of various risk factors on the primary patency. Significance was considered when the p-value was less than 0.05.

## Results

### Patients

The demographic characteristics of patients are summarized in Table 1. In total, 149 limbs in 135 patients, of 56 men and 79 women with ages ranging from 26 to 93 years (mean 63 years) were included. The symptom duration ranged from 0 to 87 days with a median of 4 days, and symptoms included leg swelling (n = 124), leg pain (n = 40), and color change (n = 5). The stage based on the symptom onset was acute in 113 patients (83.7%), and subacute to chronic in 22 (16.3%).The affected side of IFDVT was left on 87 patients, right on 34 patients, and both on 14 patients. The extent of IFDVT was iliac vein only in 12 patients (9.0%), ilio-femoral vein in 49 (36.0%), and ilio-femoro-popliteal vein in 74 (55.0%), and inferior vena cava (IVC) involvement was present in 24 (18.0%), in which case additional stent deployment was performed in the involvement segment of IVC. The cause of IFDVT were unknown in 57 patients (42.2%), compression or invasion of the vein by cancerous mass in 57 (42.2%), and May-Thurner syndrome in 21 (15.6%), respectively. Concurrent PTE was observed in 68 patients (50.4%), 15 of whom (11.1%) complained associated symptoms such as dyspnea and chest pain. The median follow- up periods of the patients was 117 days (range, 1–3374 days). At the time point of data collection, 20 patients (14.8%) were alive, 64 (47.4%) expired, and 51 (37.8%) were lost to follow-up. The causes of death in the 64 patients were progression of cancer in 45 (64.3%), infection in 7 (10.9%), respiratory failure in 3 (4.7%), bleeding in 1 (1.6%), and unknown in 7 patients (10.9%). In addition, histologic type of cancer in 91 patients with available pathologic results are listed in Table 2, excluding the other 44 patients in whom diagnosis was made based on only CECT findings and tumor markers.

**Table 1:** Patient characteristics.

Factors	Number of patients (%)
Included patients (limbs)	135 (149)
Sex ratio (M:F)	56:79
Age (mean, range)	63 (26–93)
Thrombus stage	
Acute to subacute	72/135 (53.3)
Subacute to chronic	63/135 (46.7)
Affected side	
Left	87 (64.4)
Right	34 (25.2)
Both	14 (17.8)
Location	
Iliac only	12 (8.9)
Ilio-femoral	49 (36.3)
Ilio-femoro-popliteal	74 (54.8)
IVC extension	24 (17.8)
Cause of DVT	
Unknown	57 (42.2)
Compression/invasion by cancer mass	57 (42.2)
May-Thurner syndrome	21 (15.6)
Symptoms of DVT	
Leg swelling	124 (91.9)
Pain	40 (29.6)
Color change	5 (3.7)
Symptom duration, mean (range)	8.57 (0–87)
PTE	68 (50.4)
Symptomatic	15 (11.1)
Patient status	
Alive	20 (14.8)
Expired	64 (47.4)
Lost to follow-up	51 (37.8)
Procedures	
Mechanical thrombectomy only	41 (30.4)
Mechanical thrombectomy + PTA	13 (9.6)
Mechanical thrombectomy + PTA + stent	81 (60.0)
IVC filter	130 (96.3)
Thrombolysis	12 (8.9)

DVT; deep vein thrombosis, PTE; pulmonary thromboembolism, PTA; percutaneous transluminal angioplasty, IVC; inferior vena cava.

**Table 2:** Diagnosis of 135 cancer patients based on clinical and histologic type with available pathologic results.

Clinical and image-based diagnosis (tumor marker & CT)	n = 44
Pathologic diagnosis based on confirmed histologic type	n = 91
Adenocarcinoma	47
Squamous cell carcinoma	12
Lymphoma	6
Sarcoma	5
Melanoma	4
Urothelial cell carcinoma	4
Hepatocellular carcinoma	3
Leukemia	2
Invasive ductal carcinoma of breast	2
Cholangiocarcinoma	1
Malignant meningioma	1
Mucinous carcinoma	1
Papillary carcinoma	1
Signet ring cell type	1
Small cell carcinoma	1

CT; computed tomography.

### Procedures

Among the 135 patients, 41 patients (30.4%) were only treated by aspiration thrombectomy, while additional PTA was performed in 13 patients (9.6%) and PTA/stent in 81 patients (60.0%) including all 21 patients with May-Thurner syndrome. Although the size of device was determined according to each patient’s condition, 14 or 16 mm balloons and 16 or 18 mm stents were mainly used. The outcomes of patients regarding the treatment options are summarized in Table 3. Whether the adjunctive procedure was performed did not affect clinical success (78.0% vs. 72.3%; p = 0.496), but technical success was significantly higher in the patients in whom PTA and/or stent deployment was performed compared to those did not undergo PTA/stent (78.0% vs. 95.7%; p = 0.005). IVC filter was implanted in 130 patients (96.3%) at the beginning of the procedure to prevent PTE. None of the IVC filters were retrieved, and IVC filter-related thrombosis was not detected.

**Table 3:** Summary of outcomes based on treatment.

	MAT only (n = 41)	MAT+PTA (n = 13)	MAT+PTA+stent (n = 81)	p-value
**Technical success (%)**	32 (78.0)	13 (100)	77 (95.1)	0.005
**Clinical success (%)**	32 (78.0)	8 (8)	60 (74.1)	0.496
**Recurrence (%)**	5 (12.2)	2(15.4)	19 (23.5)	0.307
**1-year primary patency (%)**	43.5	41.5	44.3	0.751

MAT; mechanical aspiration thrombectomy, PTA; percutaneous angioplasty.

### Outcomes

**Fig 1:**
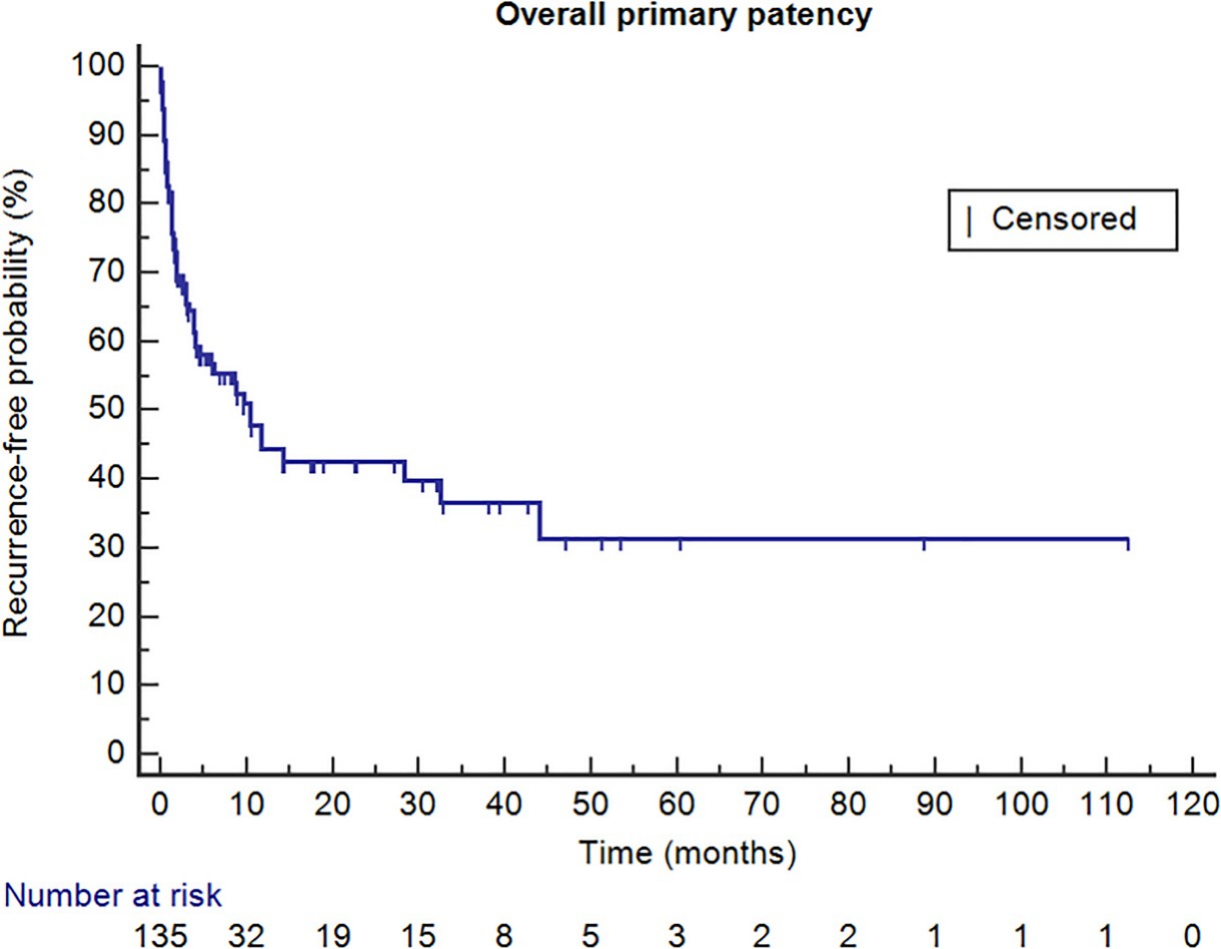
Overall primary patency.

**Fig 2:**
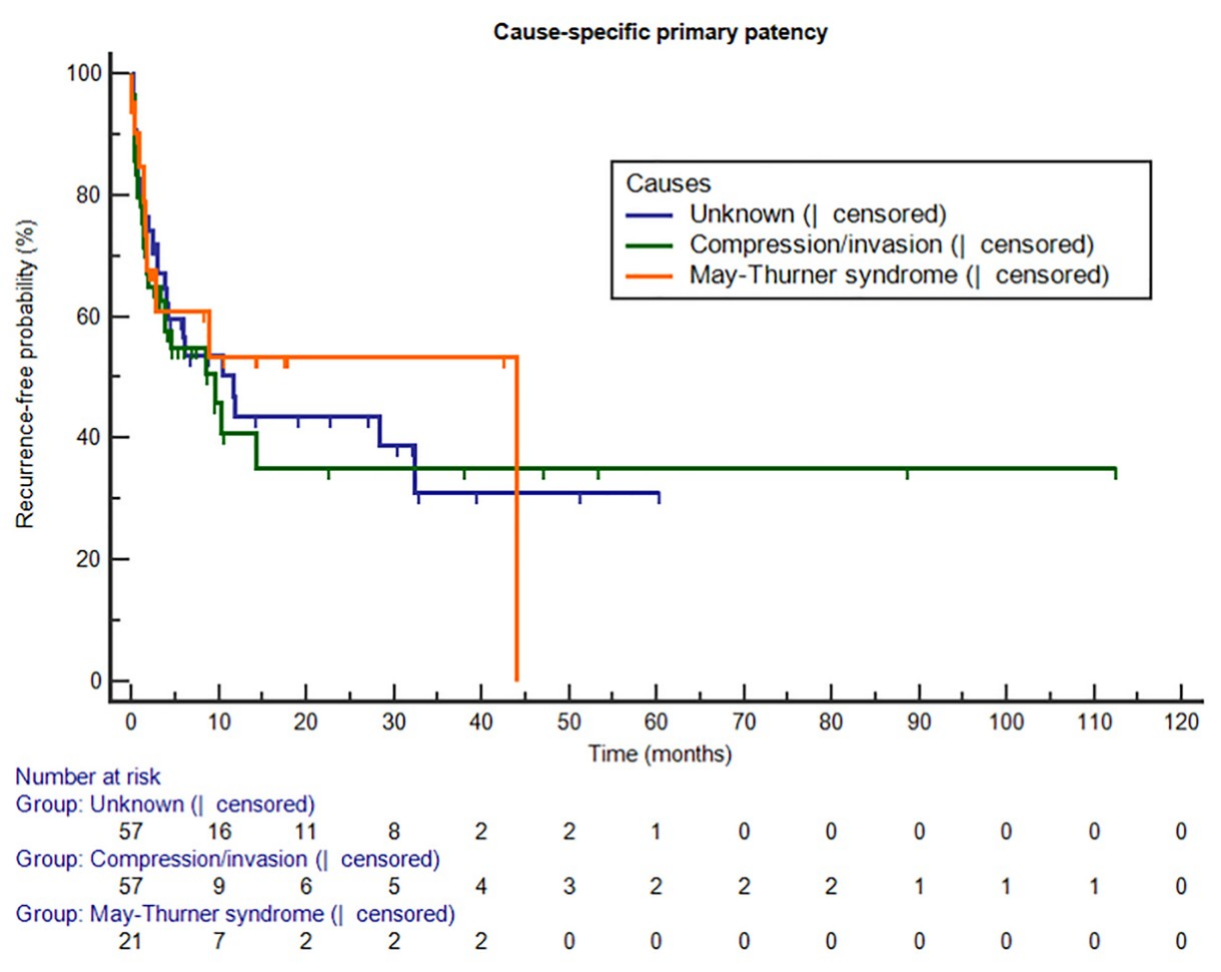
Cause-specific of primary patency.

Table 4 summarizes overall and stage-based outcomes of the procedures, and the Kaplan-Meier curve representing overall and cause-specific of primary patency are presented in Figs 1 and 2, respectively. Technical and clinical success was achieved in 89.6% and 71.5%, respectively. The overall primary patency rates of 135 patients were 88.1%, 81.6%, 76.0%, 74.1% and 69.1% at 1, 3, 6, 12, and 30 months, respectively. In subgroup analysis based on stage of IFDVT, though statistically insignificant, lower technical success was seen in patients with longer symptom duration (p = 0.180). In contrary, clinical success was similar regardless of symptom duration (p = 0.892). Recurrence of IFDVT was noted in 26 patients (19.3%) and 0.79 cases per patients-years, and results of subgroup analysis with regard to stage of IFDVT demonstrated no significant relationship between recurrence rate and symptom duration (p = 0.381). Recurrence was not significantly related to the extent of thrombus (p = 0.775), whether additional stent deployment was performed or not (p = 0.131). The primary patency rate at 1 year in overall was 74.1%, while the rates were 75.9%and 48.6% in acute and subacute to chronic stage of IFDVT, respectively.

**Table 4:** Summary of outcomes based on stage.

	Overall (n = 135)	Acute (n = 113)	Subacute to chronic (n = 22)
**Technical success (%)**	122 (90.4)	104 (92.0)	18 (81.8)
**Clinical success (%)**	100 (71.5)	83 (73.5)	17 (77.3)
**Recurrence (%)**	26 (19.3)	20 (17.7)	6 (27.3)

Acute: <14 days, subacute to chronic: ≥ 14 days.

**Fig 3:**
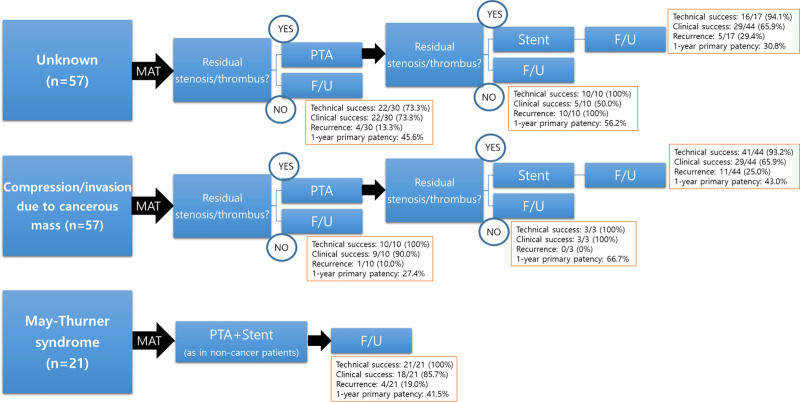
Treatment algorithm and summery of outcomes based on the cause of ilio-femoral deep vein thrombosis (IFDVT).

The results of subgroup analysis of outcomes based on the cause of IFDVT were summarized in Table 5 and Fig 3, demonstrating no significant difference in outcome parameters in all 3 groups.

**Table 5:** Subgroup analysis of outcomes based on the cause of ilio-femoral deep vein thrombosis (IFDVT).

	Cause of IFDVT			
	Unknown (n = 57)	Compression/invasion by cancerous mass (n = 57)	May-Thurner syndrome (n = 21)	p-value
**Technical success (%)**	48 (85.2)	54 (94.5)	20 (95.2)	0.179
**Clinical success (%)**	41 (71.9)	41 (71.9)	18 (85.7)	0.404
**Recurrence (%)**	11 (19.3)	12 (21.2)	3 (14.3)	0.798
**1-year primary patency (%)**	72.4	77.0	73.9	0.691

IFDVT, ilio-femoral deep vein thrombosis.

There were no procedure-related complications. Advanced age was the only statistically significant predictor of VTE recurrence (p = 0.007). None of the other assessed risk factors was related to recurrence by chi-square test (male sex; p = 0.25, recent surgery; p = 0.44, coagulopathy; p = 0.32, immobilization; p = 0.53, recent chemotherapy; p = 0.32, hypertension; p = 0.54). The mean decrease in Hb/Hct was about 0.93±1.41 (g/dL)/2.67±4.05 (%) and pre-procedural mean D-dimer level was 16.8±2.43 mg/L (normal range: < 0.5mg/L).

## Discussion

The strong positive correlation between VTE and cancer is well-known, with about 7-fold increase in the risk of VTE in cancer patients, and VTE is known to be the second leading cause of death in cancer patients [17]. Nevertheless, the value of aggressive treatment of IFDVT in patients with malignancy has been underestimated, regarding their short life expectancy as a relative contraindication and neglecting the patients’ suffering from persistent severe symptoms caused by IFDVT [13]. Management of IFDVT in cancer patients should be considered important nowadays, because survival is prolonged as treatment of malignancy has advanced and optimization of life quality of these patients is important.

The technical and clinical success rates and primary patency at 1 year in this study were 90.4%, 71.5%, and 74.1%, while the reported rates in the literature which mostly included patients without cancer were about 96–100%, 100%, and 77–96%, respectively [12,18–20]. The modest outcomes in the current study are probably owing to the fact that all subjects were cancer patients, with 16.3% of them presented with subacute to chronic stage of IFDVT. In contrast, the majority of subjects in the previously published data were without cancer and most studies included only acute stage of IFDVT. It is widely accepted that chronic thrombi are more difficult to be removed compared to acute thrombi. Park et el. demonstrated the lower technical success of MAT in subacute IFDVT of 62.5%, as compared to 96.6% in acute IFDVT group, and the same is true for thrombolysis therapy [20]. In another investigation by Oguzkurt et al., in which 21% of the study group was comprised of subacute IFDVT, thrombus removal less than 95% was achieved in 33.8%, necessitating additional CDT in 27.3% of patients [19]. Considering these facts, the patients with various stages of IFDVT in this current study were treated accordingly with aids of adjunctive procedures including PTA for thrombus maceration with or without stent deployment. The primary patency rate at 1 year follow-up in this study was 74.1%, which is better than the previous studies with primary patency rates of 68.8% and 57% in malignant and non-malignant IFDVT, respectively [21,22]. The reason for the superior outcome of the current study is not definite, but it is probably owing to the fact that the mainstay of treatment was MAT, in which a large amount of thrombi were physically removed. This result may support the need for aggressive treatment of IFDVT in cancer patients. In subgroup analysis of outcomes based on the cause of IFDVT, in which the causes were classified as unknown, compression by mass, and May-Thurner syndrome, there was no significant difference in technical and clinical success, recurrence, and 1-year primary patency among the three groups. These results may suggest that more aggressive treatment of symptomatic DVT in cancer patients is encouraged regardless of the cause.

Recurrence of VTE was seen in 26 patients (19.3%) and 0.79 cases per patient-years, regardless of the initial cause of IFDVT. A report on Japanese cohort described the rate of recurrent VTE to be 7.2 per 100 patient-years in general, and the presence of malignancy significantly increases the rate of recurrent VTE, specifically with hazard ratio of 10.7 (95% CI: 3.5–32.8) and an estimated 15% risk of recurrence per year [6,16], caused by hypercoagulable state due to malignancy [12]. According to Noble et al., VTE needs to be considered as a chronic disease as there always exists a risk of recurrence, and the cumulative incidence of recurrent VTE was 20.7% in cancer patients as compared with 6.8% in patients without cancer [17]. The recurrence rate in the current study is comparable to the results in the literature, and the higher recurrence rate of IFDVT in cancer patients necessitates caution and close surveillance to minimize recurrence.

Potential disadvantages of MAT is that it requires the use of large vascular sheath and guiding catheters, which might cause bleeding at the vascular access site and injury of the vessel wall and valves, and aspiration of large amount of thrombi with blood might cause decreased in Hb level [8,12,23]. However, no procedure-related complications including bleeding at the puncture site were observed in this study, and the mean decrease in Hb/Hct was only about 0.93±1.41 (g/dL)/2.67±4.05 (%).

In addition, concerns about complications by other forms of VTE treatment is not necessary, including hemolysis and hemoglobinuria posing risk of renal failure, bradycardia, and hypotension cause by expensive pharmacomechanical thrombolysis devices such as Angiojet [8,12,18,24]. Especially, potential bleeding complication of anticoagulation therapy and CDT is more likely to occur in cancer patients, with 2.2-fold increase in the incidence of major bleeding [17]. In contrast, MAT is beneficial for treating IFDVT in cancer patients due to its lack of bleeding risk [8,12,20].

There are several limitations in this study. First, since it is a single-center retrospective study, potential recall and selection bias might be inevitable. Second, clear distinction between the stages of IFDVT was attempted with symptom onset but it is controversial, because prediction of thrombus age is difficult and symptom duration may not represent the chronicity [24,25] and thrombi could be mixed in various stages [20]. Third, the initial diagnosis and recurrence of VTE was mainly based on the patients’ subjective symptom followed by presence of IFDVT on CECT, underestimating the importance of asymptomatic VTE. Fourth, quantitative measurement of clinical success based on symptom scale was not investigated as the current study was retrospective in design. In addition, the actual incidence of recurrence could be higher than the results presented in this study, since symptoms related to VTE could be masked by cancer itself and other more devastating clinical problems. Heterogeneity of the cohort due to various histopathology and origin of the underlying cancer is another limitation of the study. In addition, follow-up was lost in 37.8% of patients for unknown reasons, possibly due to death or abandonment of further treatment, which could not be avoided as the study included cancer patients. The loss of follow-up in many of these patients could affect the statistical power in this study. Also, comparison between cancer and non-cancer group was not performed.

In conclusion, MAT is feasible approach to treat symptomatic IFDVT in cancer patients, revealing satisfactory outcomes with minimal risk of complication.

## Supporting information

S1 Data DVT data 202106. (XLSX)
